# Spatially Formed Tenacious Nickel-Supported Bimetallic Catalysts for CO_2_ Methanation under Conventional and Induction Heating

**DOI:** 10.3390/ijms24054729

**Published:** 2023-03-01

**Authors:** Daniel Lach, Błażej Tomiczek, Tomasz Siudyga, Maciej Kapkowski, Rafał Sitko, Joanna Klimontko, Sylwia Golba, Grzegorz Dercz, Krzysztof Matus, Wojciech Borek, Jaroslaw Polanski

**Affiliations:** 1 Centre for Materials and Drug Discovery, Institute of Chemistry, Faculty of Science and Technology, University of Silesia, Szkolna 9, 40-006 Katowice, Poland; 2 Scientific and Didactic Laboratory of Nanotechnology and Material Technologies, Faculty of Mechanical Engineering, Silesian University of Technology, Konarskiego 18a, 44-100 Gliwice, Poland; 3 Institute of Physics, Faculty of Science and Technology, University of Silesia, 75 Pułku Piechoty 1a, 41-500 Chorzów, Poland; 4 Institute of Materials Engineering, Faculty of Science and Technology, University of Silesia, 75 Pułku Piechoty 1a, 41-500 Chorzów, Poland; 5 Materials Research Laboratory, Faculty of Mechanical Engineering, Silesian University of Technology, Konarskiego 18a, 44-100 Gliwice, Poland; 6 Department of Engineering Materials and Biomaterials, Faculty of Mechanical Engineering, Silesian University of Technology, Konarskiego 18a, 44-100 Gliwice, Poland

**Keywords:** CO_2_ methanation, bimetallic catalyst, Ni-wool support, Ni-mesh support, Au, Pd, Re, Ru nanoparticles, spatial and tenacious form, induction heating

## Abstract

The paper introduces spatially stable Ni-supported bimetallic catalysts for CO_2_ methanation. The catalysts are a combination of sintered nickel mesh or wool fibers and nanometal particles, such as Au, Pd, Re, or Ru. The preparation involves the nickel wool or mesh forming and sintering into a stable shape and then impregnating them with metal nanoparticles generated by a silica matrix digestion method. This procedure can be scaled up for commercial use. The catalyst candidates were analyzed using SEM, XRD, and EDXRF and tested in a fixed-bed flow reactor. The best results were obtained with the Ru/Ni-wool combination, which yields nearly 100% conversion at 248 °C, with the onset of reaction at 186 °C. When we tested this catalyst under inductive heating, the highest conversion was observed already at 194 °C.

## 1. Introduction

Excess anthropogenic CO_2_ emission gave rise to novel sustainable chemistry and engineering ideas. Power-to-gas is an example of such a concept that targets CO_2_ mitigation by using surplus energy, particularly renewable energy, to generate hydrogen, for example, from the hydrolysis of water and a further reaction of this hydrogen with carbon dioxide [1]. The main product is methane, which we can use as a synthetic natural gas (SNG). The rising prices of natural gas additionally make the concept highly attractive. The crucial reaction of the process is CO_2_ methanation (CO_2_ + 4H_2_ ⇆ CH_4_ + 2H_2_O). This reaction is discussed in detail in [2,3]. However, the practical course of CO_2_ hydrogenation to CH_4_ is impeded by many side processes, which depend on the reaction conditions. Therefore, to improve the selectivity and yield of this reaction and reduce the costs of the process, it is necessary to search for new high-performance and low-temperature catalysts.

Nickel-based catalysts are an essential class for CO_2_ methanation [3,4,5,6]. The nickel catalyst was already used in the pioneering research on the hydrogenation of carbon oxides to methane by Paul Sabatier and Jean B. Senderens in 1902 [7]. It is characterized by a high selectivity to methane and is often a good compromise between high catalytic activity and low price. The methanation mechanism on the surface of Ni catalyst [8,9,10], the influence of support [11], and the synergies between Ni and other metals or promoters [3,4,5,6] were broadly investigated. It was also noted that the Ni catalyst in CO_2_ methanation may be deactivated as a result of the formation of mobile nickel subcarbonyls due to the interaction of metal particles with the formed or temporarily present CO [9]. Therefore, one of the critical treatments to improve catalytic activity is surface modifications that allow for rapid removal of the surface nickel carbonyl species by surface-dissociated hydrogen. This process is promoted by defecting the Ni surface, which can act as the trap for hydrogen surface transport, reducing the activation energy of hydrogen dissociation [10]. For example, such a mechanism was proved by the high-activity CO_2_ methanation with the sponge Ni-catalyst, which has many fcc-Ni crystal defects [12]. Due to their excellent mass and heat transfer efficiency, nickel foams are attractive as a substrate for microstructural catalysts, especially for highly exothermic reactions such as methanation. The desirable mechanical strength, high surface area to volume, and low flow pressure drop in the fixed bed reactor are also advantages. This fact was used by the authors of the composite catalyst Ni-Al_2_O_3_/Ni-foam [13] and Ru/CeO_2_/Ni-foam [14]. tenacious catalyst produced by the wash-coating method with the Ni/CeO_2_ component on an aluminum honeycomb bed was also presented in [15]. Another example of the Ni-based formed catalysts is a quaternary disc-shaped system (made of Ni, Ti, Ce, and yttria-stabilized zirconia (YSZ)) [16]. For the above examples, the conversion to methane at 250 °C varies between 15% and 65%. The preparation method is often multi-stage, energy- and time-consuming, and requires several constituent materials. There are already high-efficiency and low-temperature catalysts for CO_2_ methanation, but in the form of grains or nickel nanowires. For example, we presented such materials in [17,18]. However, there have been no reported attempts to prepare tenacious and compact nickel-based spatial catalysts with satisfactory results for potential commercialization, maintaining high-performance and low-temperature catalysis in CO_2_ methanation.

This article presents a novel approach to preparing bimetallic catalysts with spatially formed tenacious Ni-support based on mesh or wool. The supports were combined with nano -Au, -Pd, -Re, or -Ru. Nanometals for impregnation were generated using our recently developed method for powder catalysts [17,18,19,20,21]. This method minimizes the use of an expensive catalyst component in bimetallic conjugation. In addition, our new catalyst support formation procedure could be easily scaled to a commercial product. The Ru/Ni-wool combination achieved the best result, which provides almost 100% conversion in CO_2_ methanation at 248 °C with the onset of reaction at 186 °C. The best sample was also tested in a methanation reactor with induction heating. For such a system, the highest conversion was noted already at 194 °C.

## 2. Results and Discussion

### 2.1. The Catalysts Design, Preparation, and Structure

**Figure 1:**
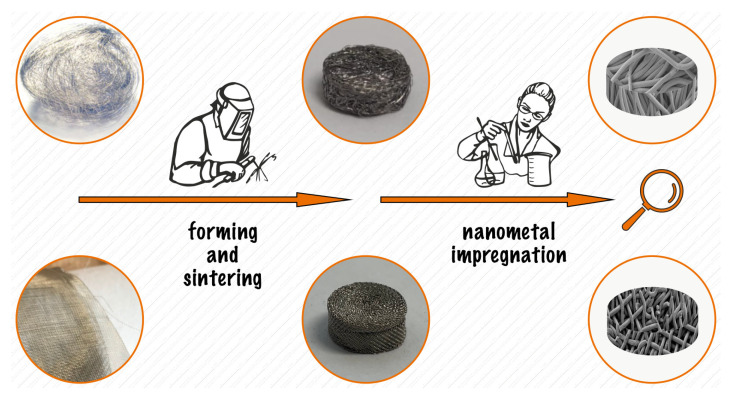
Scheme of the preparation procedure for Ni-wool or Ni-mesh supported catalysts.

**Figure 2:**
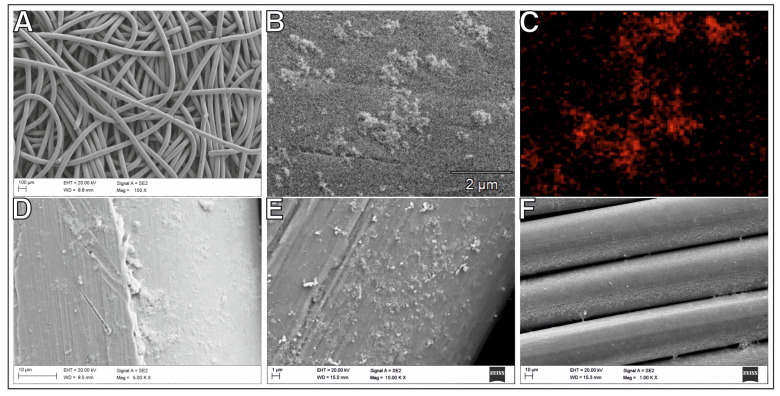
Scanning electron microscopy (SEM) images for nanometal/Ni-wool catalysts;(**A**) Ni-wool support fibers with Ru nanoparticles, (**B**) Ru nanoparticles on the support surface, (**C**) Ru nanoparticles (red spots) after EDS mapping, (**D**) Re nanoparticles on the fiber surface, (**E**) Pd nanoparticles on the fiber edge surface, (**F**) support fibers covered with Au nanoparticles.

**Figure 3:**
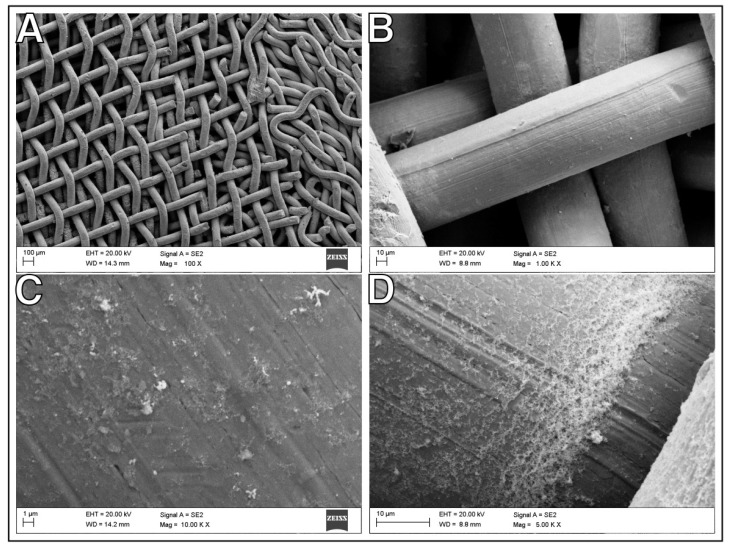
Scanning electron microscopy (SEM) images for nanometal/Ni-mesh catalysts, (**A**) Ni-mesh support fibers with Ru nanoparticles, (**B**) Re nanoparticles on the fibers surface, (**C**) Pd nanoparticles on the fiber surface, (**D**) Au nanoparticles on the surface of the fibers in contact.

**Figure 4:**
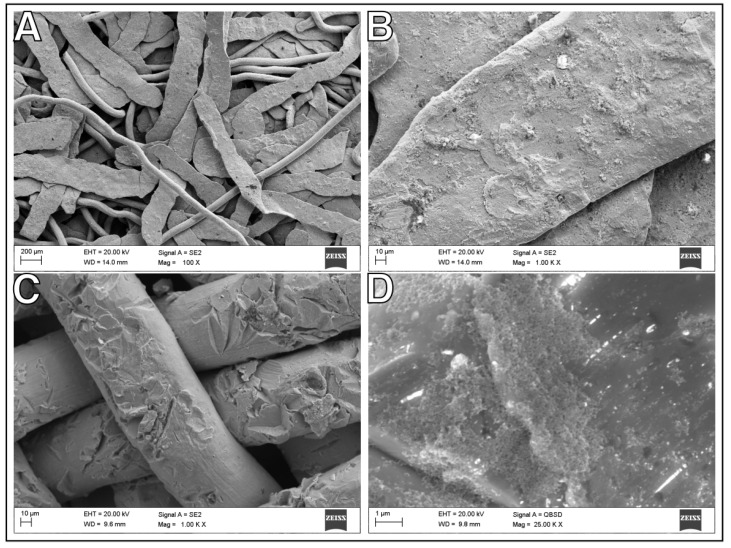
Scanning electron microscopy (SEM) images for nanoRu/Ni-ground_wool and nanoRu/Ni-blasted_mesh catalysts, (**A**) support fibers of ground nickel wool ornamented with naonRu, (**B**) Ru nanoparticles on the surface of ground fibers, (**C**) support fibers of sandblasted nickel mesh ornamented with naonRu, (**D**) Ru nanoparticles on the surface and in the pits of Ni-blasted_mesh fibers.

**Figure 5:**
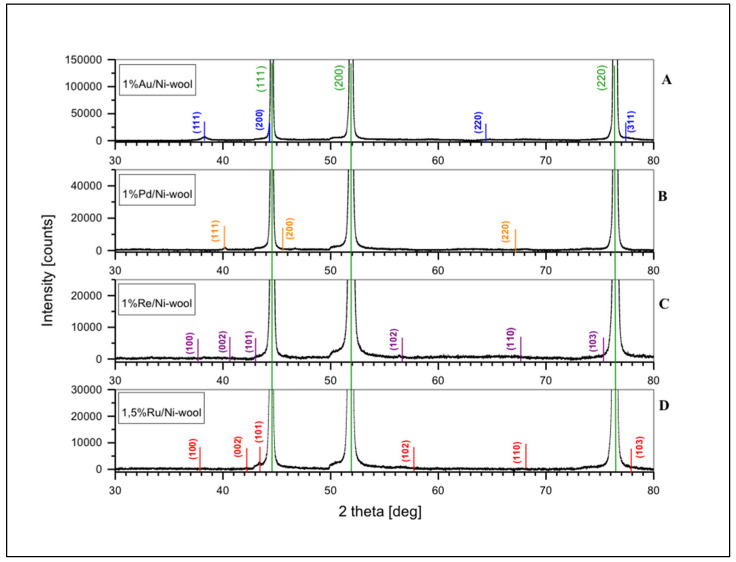
X-ray diffraction patterns at 2θ: 30°–80° for 1.0% Au/Ni-wool (**A**), 1.0% Pd/Ni-wool (**B**), 1.0% Re/Ni-wool (**C**), 1.5% Ru/Ni-wool (**D**) samples. Miller indices for experimental peaks of Ni (green) wool fibers and Au (blue), Pd (orange), Re (purple), Ru (red) metals are marked.

Multi-component materials are commonly used in engineering to improve catalyst performance [22,23]. A typical representative of a heterogeneous catalyst consists of a metal and a support in the form of oxides (e.g., SiO_2_, Al_2_O_3_, TiO_2_), zeolites, carbon, or metaloorganic compounds [6,11,24,25]. Maximizing the metal surface area for a specific metal weight is essential in optimizing the catalyst [26]. Therefore, the small metal particles (typically less than 1–10 nm) are synthesized, and anchored to a thermally stable, high-surface-area support. However, the final catalytic material is often in powder or non-solid form. This form is not particularly commercially valuable. Scaling up is also a typical problem for such a catalyst form. Relatedly, we formulated a tenacious spatial catalyst consisting of commercially available nickel wool or mesh and enriched with selected metal nanoparticles: Au, Pd, Re, Ru. A scheme of the preparation procedure is shown in Figure 1. The obtained materials were tested as candidates for CO_2_ methanation catalysts. The morphology and composition of the resulting bimetallic system were studied using scanning electron microscopy (SEM) (Figure 2, Figure 3 and Figure 4), specific surface area (SSA) (Table 1), X-ray diffraction spectroscopy (XRD) (Table 2 and Figure 5), and energy-dispersive X-ray fluorescence spectrometry (EDXRF) (Table 3). Additional materials from the analyses are included in the Supplementary Materials.

Two types of nickel support were made. The first was made of rolled up nickel mesh and impulse sintered. The second was formed from nickel wool, which was compressed and impulse sintered. Pictures of these materials are shown in Figure 1, Figure 2 and Figure 3, and in the Supplementary Materials (Figures S1–S3). In our previous study of a CO_2_ methanation catalyst supported by nickel nanowires, we emphasized the significant effect of the extended surface area of the catalytic material [18]. Here, we also tried to improve the specific surface area of the presented supports. Modifications were made by ball milling wool and in the case of mesh by sandblasting. The specific surface area (SSA) of support materials is given in Table 1. The specific surface area of the Ni-mesh support is larger than that of Ni-wool. The difference in the diameter of the nickel wire in both cases decides this result. For the mesh wire, the lateral surface of the cylinder (with the same height compared) is almost 50,000 nm^2^ larger. The milling process increased the SSA of the wool-type threefold. In the second modification, sandblasting did not improve the SSA of Ni-mesh and even lowered it according to the S bet analysis. Although SEM images (Figure 4) show furrows, pits, and roughness, we hypothesize that the walls of the grooves and irregularities have been smoothed out, hence, the failing of increased SSA. Nevertheless, SSA improvement research still needs to continue.

As we have already mentioned, in the preparation of a heterogeneous multi-component catalyst, in this case a bimetallic one, it is important to obtain a narrow size distribution of metallic nanoparticles and their large dispersion on the support. This feature was achieved using our proprietary method of synthesis of metal nanoparticles on a silica matrix, then digestion of the matrix with sodium hydroxide and uniform suspension of nanoparticles in the impregnation solution for support coverage. We described this method in [17]. Nanoparticles on silica with an average size of 4.1 nm for Ru, 4.4 nm for Pd, 5.1 nm for Au, and 1.8 nm for Re were used. Transmission electron microscope (TEM) images of the nanoparticles and their size distribution are given in the Supplementary Materials (Figures S4–S8). The structure of the catalyst material after ornamentation with nanoparticles of selected metals is shown in Figure 2, Figure 3 and Figure 4, and in the Supplementary Materials (Figures S1–S3). The material forms a conglomerate of mesh or wool fibers with metal particles Au, Pd, Re, Ru, respectively. The nanometal coating on the substrate fibers is distributed over the entire surface in a non-uniform manner. Metal aggregation on the fibers is visible, in particular at the crossing of the support wires. The concentration of metal nanoparticles can also be seen in any imperfections or scratches on the surface of the nickel fibers. Microscopic examinations proved that the distribution of particles strongly depends on the surface roughness of the nickel support. Different shape and size of the nanoparticles are observed depending on the selected metal. The size and lattice parameters of the nanometal particles were determined using the XRD technique. We used the Scherrer equation to estimate the average crystalline particle size from the highest intensity diffraction peaks. The measured values of metal nanoparticles range from about 6 nm to 12 nm. Lattice parameters and average crystallite dimensions (D) are listed in Table 2. For compositions with the best-performing support (Ni-wool), the XRD spectra are shown in Figure 5.

The X-ray diffraction patterns of the 1%Au/Ni-wool, 1%Pd/Ni-wool, 1%Re/Ni-wool, and 1.5%Ru/Ni-wool are given in the range of the 2θ angle from 30 to 80 degrees. They clearly show the diffraction lines that correspond to the face-centered cubic (Fm3m) phase of Ni (JCPDS 01-077-8341), whereas only the most intense peaks of the cubic (Fm3m) phases of Au (2θ_111_~38°) and Pd (2θ_111_~40°) are identified. The overlapping diffraction lines were observed. The strongest diffraction lines of the hexagonal (P63/mmc) phases of Ru and Re (2θ_101_~43°−44°) overlap Ni (111) diffraction line, whereas the less intensive peaks of Ru and Re were not detected. The qualitative and quantitative elemental analysis was performed by EDXRF spectrometry. The results of the quantitative analysis calculated by the fundamental parameter method are presented in Table 3. The content of nanometal in the sample was up to 1%. This percentage is the optimal support load as studied in [27] and is consistent with our experience and testing Above this concentration, we observed either a complete coverage of the support fibers or an agglomeration, which increased the size of the nanoparticles. These effects reduced the number of nanometal-support connections (synergy centers between materials), decreasing catalyst activity.

### 2.2. The Catalysts in CO_2_ Methanation

**Figure 6:**
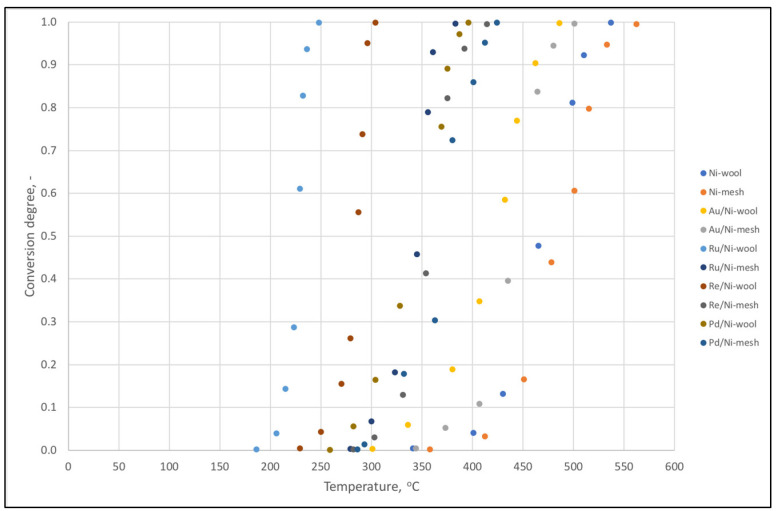
CO_2_ conversion of catalysts made of nickel wool or nickel mesh and ornamented with ca. 1% nano-Ru, -Re, -Pd, or -Au.

Kinetic limitations affecting the hydrogenation of carbon dioxide to methane with an acceptable rate and selectivity necessitate the use of a catalyst [3]. The set of catalytic materials presented above was tested in relation to methane conversion during a temperature increase, as shown in Figure 6. The best Ru/Ni-wool composition was determined. It achieves almost 100% conversion at 248 °C, with the onset of reaction at 186 °C. This composition is consistent with our previous research [17,18,19] and confirms the privilege of the Ru/Ni connection in CO_2_ methanation catalysis, which we wrote about in [28]. Approximately 100% conversion of the best composition in relation to the reference sample-pure nickel wool support is possible at a temperature lower by as much as 289 °C. Compared to the previously studied CO_2_ methanation catalysts, such as Ru/Ni-nanowires, Ru/Ni-grains, almost complete conversion of a mixture of 20% CO_2_ and 80% H_2_ to methane at a flow rate 3 dm^3^/h is for temperatures as follows: Ru/Ni-nanowires 179 °C, Ru/Ni-grains 204 °C, Ru/Ni-wool 248 °C. For powder catalysts, weight hourly space velocity (WHSV) was equal to 6.5 h^−1^, and for the present sample it was 1.8 h^−1^. In turn, for example, for the most similar, tenacious, and spatial materials, the conversion at 250 °C is Ni-sponge 83% [12] and Ru/CeO_2_/Ni-foam disc ca. 15% [14]. The gas hourly space velocity (GHSV) values, calculated with inlet flow rate of CO_2_, were Ni-sponge 4200 h^−1^, Ru/CeO_2_/Ni-foam disc approx. 714 h^−1^, and Ru/Ni-wool 3612 h^−1^. The difference in performance in the case of the first comparison can be explained by the specific surface area, the number of active centers or diffusion, which advantage grains and nanowires. However, in the second case, we see a significant superiority of the obtained material over previous commensurable materials, probably thanks to the Ru-Ni synergy and differences in the adsorption of surface forms of reactants. The presented material does not use typical oxides (CeO_2_, ZrO_2_, Al_2_O_3_, SiO_2_, TiO_2_) as the support construction, and the reaction path runs only through the area of Ru and Ni atoms.

**Figure 7:**
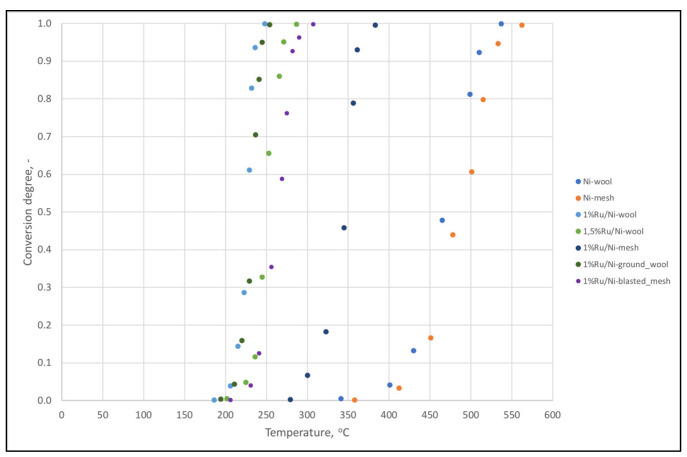
CO_2_ conversion of catalysts made of modified nickel wool, nickel mesh, and ornamented with nano-Ru.

In this research, we also attempted to modify the support surface. Results in CO_2_ methanation for the best compositions in comparison with ground or sandblasted nano-Ru support samples are shown in Figure 7. Supports crafted of Ni-wool fare much better than those of Ni-mesh. The difference in the morphology of the material can explain the observed phenomenon. The Ni-wool support is a highly irregular arrangement of fibers, which may impact a more turbulent flow of gases and a longer contact time of the reactants with the catalyst. There can be a difference in diffusion effects for both types of supports [29,30]. No significant catalytic improvement was observed for the ground nickel wool support. However, the earlier sandblasting of the mesh and the formation an irregularly layered support from its pieces increases the activity of Ru/Ni-blasted_mesh relative to Ru/Ni-mesh at about 280 °C by as much as 90%. The difference in favor of the Ru/Ni-wool at around 250 °C is 65%. The improvement of the catalyst mesh benchmark can be explained by the hydrogen traps in pits after sandblasting and structural changes in the support (see Figure 4c). These changes probably translate into increased hydrogen uptake, improved hydrogen spillover, and transport of the species adsorbed or formed on the surface of the catalytic material.

The best Ru/Ni-wool composition was tested in CO_2_ methanation for 24 h at the highest conversion temperature (248 °C). No significant decrease in catalyst efficiency was observed during this time. XRD analysis of the sample also showed no destabilization. A slight difference in the values of the lattice constants Ni (0.002 Å) was noted; however, it is within the limits of the measurement error. We performed XPS (X-ray photoelectron spectroscopy) to profile the sample before and after the reaction. Analysis of chemical states indicated the presence of oxidized forms of composition metals. Carbon species have also been detected on the surface. After methanation, a significant share of carbon bonding to oxygen was observed, mainly corresponding to the C=O bond. Spectra, measurement details, and additional descriptions are given in the Supplementary Materials (Figure S9). Long-term catalyst deactivation tests (over 24 h) have not yet been performed (subject to further research). However, we assume that the behavior of this catalytic material will be analogous to the ones we studied earlier [17,18,19], and reactivation will be feasible by hydrogen treatment. For comparative purposes, we additionally tested the best sample in a reactor with direct bed induction heating. Induced heating eliminates limitations in heat transfer in the catalyst bed and improves energy efficiency, which we wrote about in [19], and it was broadly described in [31,32]. In such a system, it was possible to decrease the initial reaction temperature to Ti = 172 °C. The conversion degree of 99.9% was reached at 194 °C as opposed to 248 °C for the conventional heating system. The exact explanation of the reason for the improvement may be a topic for a separate publication, but our hypothesis assumes the generation of eddy currents in the support filaments, which can affect the electron modification of atoms and thus the potential differences and the energy barrier to overcome by intermediates and surface moiety in the mechanism of CO_2_ methanation. The theory of hot electrons may also play a role here [33,34]. Research into scaling up and potential commercialization of the presented material is still in progress.

## 3. Materials and Methods

### 3.1. Catalysts Preparation

The catalysts were made in two steps: (1) preparation of the nickel support, (2) generation of Au, Pd, Re, or Ru nanoparticles and their subsequent ornamentation on the support surface.

#### 3.1.1. Ni Support Preparation

**Figure 8:**
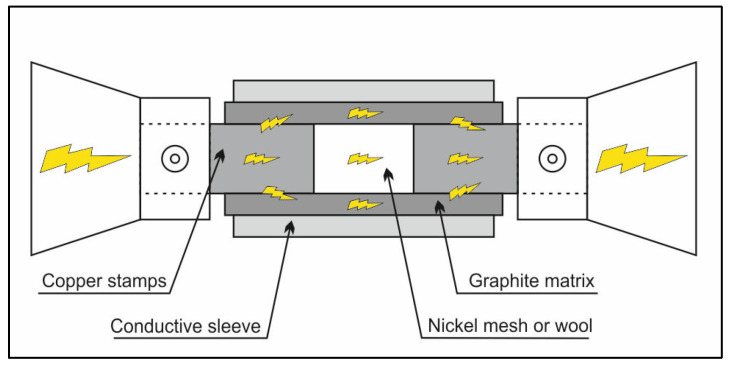
Scheme of preparation of the nickel support.

Two types of nickel support were made from commercially available materials. The first one uses the nickel wool brand “Elemental Microanalysis”. The thickness of the nickel wool wire was 0.065 mm. The second one was “Speorl KG” mesh with a wire thickness of 0.08 mm and a mesh size of 125 × 224 µm. The rolled mesh or wool was placed in a cylindrical graphite matrix and closed on both sides with copper stamps, which act as electrodes in the impulse resistance welding process. For sample formation, a thermo-mechanical simulator Gleeble-3800 from Dynamic System Inc. was used. A particular set of tools has been developed for impulse sintering of porous nickel skeletons, whose scheme is shown in Figure 8.

The die filled with nickel wool or mesh sample was placed into the Gleeble-3800 simulator. In the first step, the nickel mesh or wool were compressed to a distance of 6 mm between the copper stamps. During the initial pressing, compressive stresses of about 10–15 MPa were generated in the sample. When a vacuum of about 3 × 10^−1^ mBar was created in the Gleeble chamber, a program was started which consisted of heating the sample to a temperature of 700 °C at 10 s with further compression of the sample to a thickness value of, respectively, 3.3 mm for mesh or 3 mm for wool and with a disc diameter of 8.5 mm. During the experiment, a high electric current passes through the sample, simultaneously heating it to the pre-set temperature with at a predetermined heating rate 70 °C/s. The mass of the tested supports for each catalytic material is given in Table 3. Modified supports were prepared similarly, but the wool had been ground previously for 15 min in a planetary ball mill with 20 mm size zirconia balls. In turn, the mesh was sandblasted, cut into fragments, irregularly layered, and formed into the target disc.

#### 3.1.2. Impregnation of Ni Support with Nanometal

Metal nanoparticles were prepared according to our method described in [17]. Metal nanoparticles digested from the silica precursor with 40% NaOH solution were washed to neutral pH and centrifuged. Then the nanoparticles were suspended in 0.7 mL of isopropyl alcohol in a sonic bath. The solution was taken into a 1 mL syringe fitted with a needle. A nanometal solution was spotted onto the previously degreased and dried nickel support. The soaked material was dried at 110 °C in an oven. The application and drying procedure were repeated until the solution was exhausted. Each time the application side of the nickel support was changed. The mass of individual nanometals deposited on the silica carrier and used after digesting for impregnation is given in Table 3.

### 3.2. Method of Catalysts Characterization

Images of the surface morphology of the studied materials were obtained with a scanning electron microscope SUPRA 35 Zeiss with EDS detector for microanalysis of chemical composition.

The quantitative and qualitative chemical composition was confirmed by energy dispersive X-ray fluorescence spectrometry (EDXRF), performed on an Epsilon 3 spectrometer (Panalytical, Almelo, The Netherlands) with an Rh target X-ray tube with 50 µm Be window and max. power of 9 W. The spectrometer was equipped with a thermoelectrically cooled silicon drift detector (SDD) with an 8 µm Be window and a resolution of 135 eV at 5.9 keV. The quantitative analysis was performed using Omnian software and was based on the fundamental parameter method and following measurement conditions: 12 kV, 50 µm Al primary beam filter, 300 s counting time, helium atmosphere for Pd and Ru determination; 30 kV, 100 µm Ag primary beam filter, 120 s counting time, air atmosphere for Ni, Re, and Au determination. The current of the X-ray tube was fixed so that it would not exceed a dead-time loss of ca. 50%.

The X-ray diffraction experiments were performed on a PANalytical Empyrean diffractometer with Cu Kα radiation (40 kV, 30 mA) equipped with a PIXcel detector. Data were collected in the 20°–100° 2θ range with 0.0131° step. A qualitative phase analysis employed the “X’Pert High Score Plus” computer program and the data from ICDD PDF-4 database. Crystal lattice parameters were calculated using the Chekcell V4 program.

The specific surface area (SSA) was determined using a Gemini VII 2390 a analyzer (Micromeritics Instruments Corp., Norcross, GA, USA) at the boiling point of nitrogen (−196 °C) using the Brunauer–Emmet–Teller (BET) method. Samples before the measurments were thermal-treated at 300 °C for 1 h to remove gases and vapors that may have adsorbed on the surface during the synthesis. This was performed with a VacPrep 061 degassing system (Micromeritics Instruments Corp., Norcross, GA, USA). Samples not analyzed immediately after the degassing procedure were kept at 60 °C. Correctness of the instrument was verified by analyzing a Carbon Black reference material of known surface area (P/N 004-16833-00 from Micromeritics, Norcross, GA, USA).

### 3.3. Methanation

The catalysts were tested in an 8 mm diameter fixed bed quartz flow reactor under atmospheric pressure. The feed mix was 20% CO_2_ + 80% H_2_ and was fed continuously at a flow rate of 3 dm^3^/h. The conversion of CO_2_ to CH_4_ was investigated by exhaust gas analysis using an on-site gas analyzer GX-6000 RIKEN and a gas chromatograph SRI 310 C equipped with a thermal conductivity detector (1/8 inch diameter, 3 m long column; micropacked with active carbon 80–100 mesh; 80 °C temperature of column with argon as the carrier gas with flow rate of 10 dm^3^/h^−1^). The methane detection limit was 1 ppm for the GX-6000 and 10 ppm for the gas chromatography SRI 310 C.

For a selected catalytic system with high activity, comparative tests were carried out by replacing thermal heating with induction heating (according to the methodology used in [19]). A 100 W induction heater was used for this purpose, keeping the other parameters unchanged (size and dimensions of the catalyst bed, substrate flows). Temperature of the gases flowing out of the catalytic bed was measured.

## 4. Conclusions

The search for new methods of carbon dioxide management increases the interest in catalytic methanation of CO_2_. In search of novel bimetallic catalyst candidates for this reaction, we developed a spatially formed tenacious Ni-support based on mesh or wool. Au, Pd, Re, Ru nanometals were selected to ornamentation the support of the tested catalysts. We developed a new method for the catalyst support formation and its impregnation with nanometals. The catalyst preparation could be easily scaled up to a commercial procedure. The obtained catalyst candidates were analyzed by SEM, XRD, and EDXRF. The best combination appeared to be the Ru/Ni-wool achieving almost 100% conversion at 248 °C, with the reaction onset at 186 °C. We also tried to modify the support surface by milling wool or sandblasting and irregular layering of the mesh. There was a 90% improvement in conversion at 280 °C for Ru/Ni-blasted_mesh compared to Ru/Ni-mesh. However, this combination had a 65% lower conversion than the best Ru/Ni-wool at 250 °C. Comparatively, we tested the activity of Ru/Ni-wool in a reactor with induction heating, which significantly improves the efficiency. For such a system, the initial reaction temperature was Ti = 172 °C and a conversion degree of 99.9% was reached at 194 °C.

## Figures and Tables

**Table 1:** Specific surface area (SSA) for the tested supports.

No	Support	S Bet [m_2_/g]
1	Ni-wool	0.104
2	Ni-ground_wool	0.338
3	Ni-mesh	0.280
4	Ni-blasted_mesh	0.097

**Table 2:** The average crystallite size and lattice parameters of the investigated catalysts as determined by the X-ray diffraction technique (XRD) method.

No.	Catalyst	Lattice Parameters [Å]	D [nm]				
			*Ni*	*Pd*	*Au*	*Ru*	*Re*
1	1%Ru/Ni-wool	a = 3.516 (±0.005) for Ni	40	-	-	6	-
2	1.5%Ru/Ni-wool	a = 3.528 (±0.003) for Ni	50	-	-	10	-
3	1%Ru/Ni-ground_wool	a = 3.516 (±0.004) for Ni	20	-	-	7	-
4	1%Ru/Ni-mesh	a = 3.519 (±0.006) for Ni	40	-	-	8	-
5	1%Ru/Ni-blasted_mesh	a = 3.530 (±0.004) for Ni	40	-	-	9	-
6	1%Re/Ni-wool	a = 3.520 (±0.003) for Ni	25	-	-	-	6
7	1%Re/Ni-mesh	a = 3.528 (±0.004) for Ni	95	-	-	-	7
8	1%Pd/Ni-wool	a = 3.524 (±0.003) for Ni a = 3.886 (±0.006) for Pd	60	12	-	-	-
9	1%Pd/Ni-mesh	a = 3.516 (±0.004) for Ni a = 3.880 (±0.005) for Pd	70	8	-	-	-
10	1%Au/Ni-wool	a = 3.523 (±0.004) for Ni a = 4.071 (±0.006) for Au	55	-	6	-	-
11	1%Au/Ni-mesh	a = 3.533 (±0.004) for Ni a = 4.079 (±0.005) for Au	60	-	6	-	-

**Table 3:** Mass of support and nanometals, and EDXRF analysis of Ru, Re, Pd, Au, and Ni for the tested catalytic materials.

No.	Catalyst	^1^ Support Mass [mg]	^2^ Nanometal Mass [mg]	Weight Percentage of a Chemical Element [wt%]				
				*Ru*	*Re*	*Pd*	*Au*	*Ni*
1	1%Ru/Ni-wool	733.42	0.211	0.94	-	-	-	97.03
2	1.5%Ru/Ni-wool	726.10	0.428	1.50	-	-	-	96.50
3	1%Ru/Ni-ground_wool	726.38	0.209	0.91	-	-	-	91.70
4	1%Ru/Ni-mesh	1034.12	0.318	0.95	-	-	-	96.50
5	1%Ru/Ni-blasted_mesh	991.11	0.132	0.69	-	-	-	96.34
6	1%Re/Ni-wool	731.78	1.727	-	0.60	-	-	97.80
7	1%Re/Ni-mesh	842.10	2.502	-	0.51	-	-	98.20
8	1%Pd/Ni-wool	716.37	0.231	-	-	0.76	-	97.00
9	1%Pd/Ni-mesh	968.40	0.214	-	-	0.76	-	96.60
10	1%Au/Ni-wool	702.60	0.202				0.88	96.09
11	1%Au/Ni-mesh	836.00	0.239	-	-	-	0.78	96.29

^1^ Mass after degreasing and drying of the support material. ^2^ Mass of individual nanometals deposited on the silica carrier and used after digesting for support impregnation.

## Data Availability

Data is contained within the article or Supplementary Materials.

## Supplementary Materials

The following supporting information can be downloaded at: https://www.mdpi.com/article/10.3390/ijms24054729/s1.
