# Supplementary material for: Spatially Formed Tenacious Nickel-Supported Bimetallic Catalysts for CO2 Methanation under Conventional and Induction Heating
Source: Int J Mol Sci. 2023 Mar 1;24(5):4729. doi: 10.3390/ijms24054729 (PMC10002539; doi:10.3390/ijms24054729)
Supplement: Supplementary file 1 [file ijms-24-04729-s001.zip › ijms-2228542-supplementary.pdf]

Spatially formed tenacious nickel-supported bimetallic catalysts for CO2 methanation under conventional and induction heating.

Daniel Lach [1,*], Błażej Tomiczek [2], Tomasz Siudyga [1,*], Maciej Kapkowski [1], Rafał Sitko [1], Joanna Klimontko [3], Sylwia Golba [4], Grzegorz Dercz [4], Krzysztof Matus [5] Wojciech Borek [6], Jarosław Polański [1]

[1] Institute of Chemistry, Faculty of Science and Technology, University of Silesia, Szkolna 9, 40-006 Katowice, Poland: maciej.kapkowski@us.edu.pl (M. K.), rafal.sitko@us.edu.pl (R. S.), jaroslaw.polanski@us.edu.pl (J. P.)
[2] Scientific and Didactic Laboratory of Nanotechnology and Material Technologies, Faculty of Mechanical Engineering, Silesian University of Technology, Konarskiego 18a, 44-100 Gliwice, Poland: blazej.tomiczek@polsl.pl (B. T.)
[3] Institute of Physics, Faculty of Science and Technology, University of Silesia, 75 Pułku Piechoty 1a, 41-500 Chorzów, Poland: joanna.klimontko@us.edu.pl (J. K.),
[4] Institute of Materials Engineering, Faculty of Science and Technology, University of Silesia, 75 Pułku Piechoty 1a, 41-500 Chorzów, Poland: sylwia.golba@us.edu.pl (S. G.), grzegorz.dercz@us.edu.pl (G. D.)
[5] Materials Research Laboratory, Faculty of Mechanical Engineering, Silesian University of Technology, Konarskiego 18a, 44-100 Gliwice, Poland: krzysztof.matus@polsl.pl (K. M.)
[6] Department of Engineering Materials and Biomaterials, Faculty of Mechanical Engineering, Silesian University of Technology, Konarskiego 18a, 44-100 Gliwice, Poland: wojciech.borek@polsl.pl (W. B.)
* Correspondence: daniel.lach@us.edu.pl (D. L.), tomasz.siudyga@us.edu.pl (T. S.)

SEM images of Fig. S1-S3 available at: https://drive.google.com/drive/folders/1ytbmwstyO6mKThJRsawwjnm60rOaIy4n?usp=sharing

Fig. S1-S3; SEM images of metal nanoparticles (Ru, Re, Pd, Au) supported by Ni-wool (S1), Ni-mesh (S2), Ni-modified_wool_or_mesh (S3).

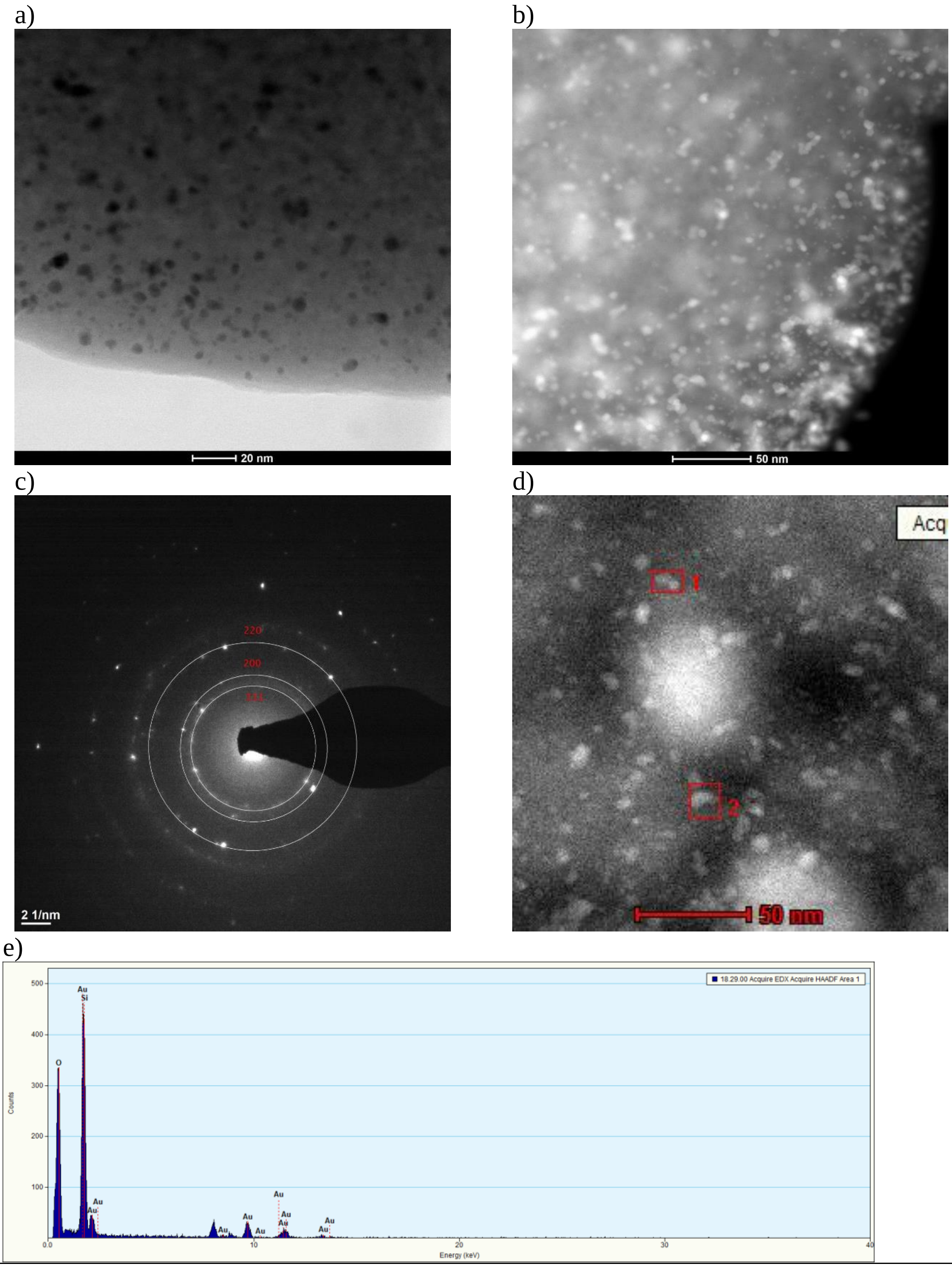

Element	Weight %	Atomic %	Uncert. %	Correction	k-Factor
-------	--------	--------	---------	----------	--------
O(K)	48.53	68.62	0.72	0.51	1.921
Si(K)	36.87	29.69	0.45	0.92	1.000
Au(L)	14.59	1.67	0.45	0.99	4.037

Fig. S4. Results of transmission electron microscopy analysis of Au nanoparticles: a) STEM-BF image, b) HAADF image, c) SAED diffraction images of selected Au particles, d,e) chemical composition analysis obtained using the characteristic EDS X-ray dispersion spectroscopy obtained in the areas indicated in the microscopic image (d)

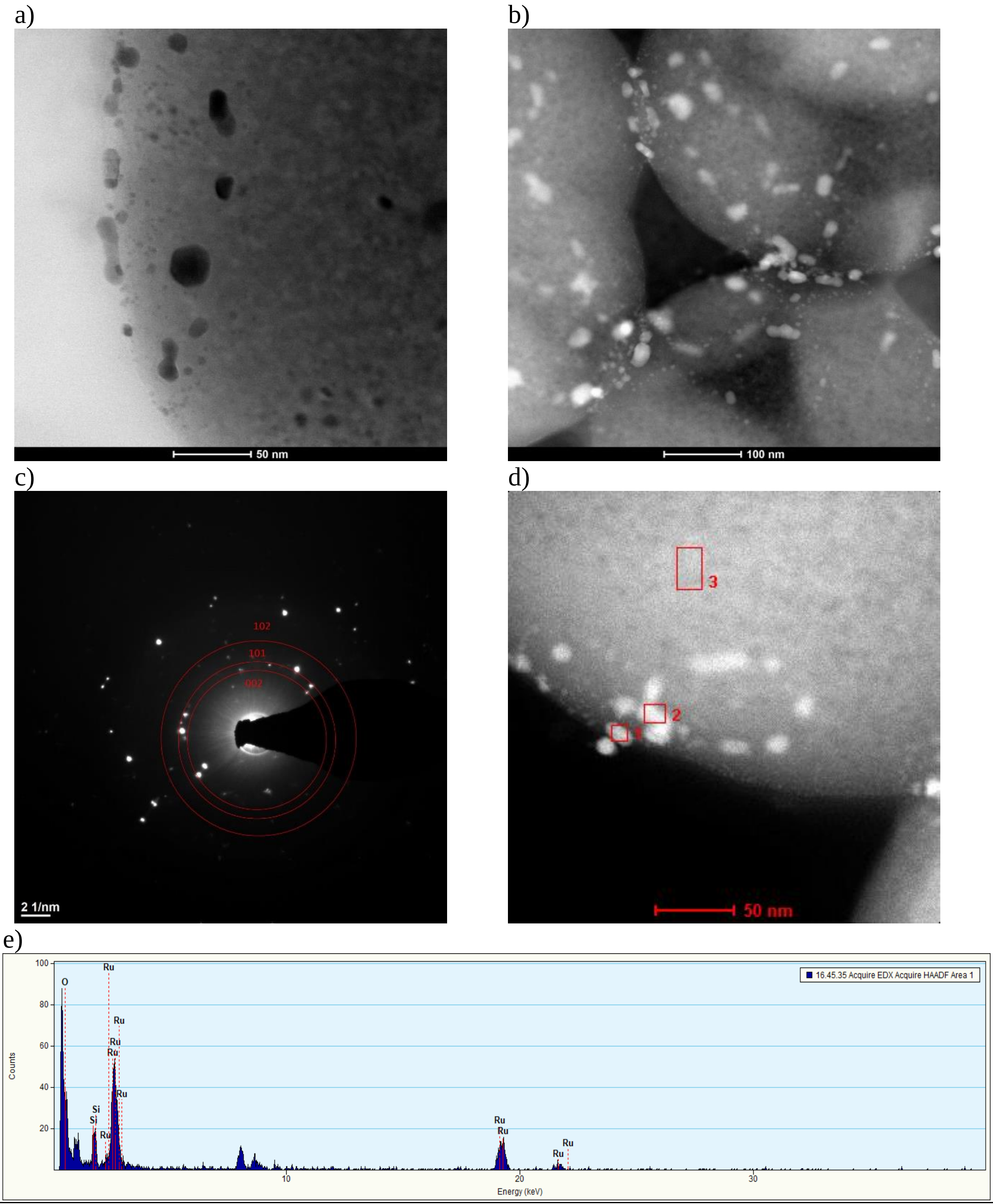

				Detector	
Element	Weight %	Atomic %	Uncert. %	Correction	k-Factor
-------	--------	--------	---------	----------	--------
O(K)	40.26	77.34	1.84	0.51	1.921
Si(K)	5.67	6.21	0.59	0.92	1.000
Ru(K)	54.05	16.43	3.24	0.96	4.813

Fig. S5. Results of transmission electron microscopy analysis of Ru nanoparticles: a) STEM-BF image, b) HAADF image, c) SAED diffraction images of selected Au particles, d,e) chemical composition analysis obtained using the characteristic EDS X-ray dispersion spectroscopy obtained in the areas indicated in the microscopic image (d)

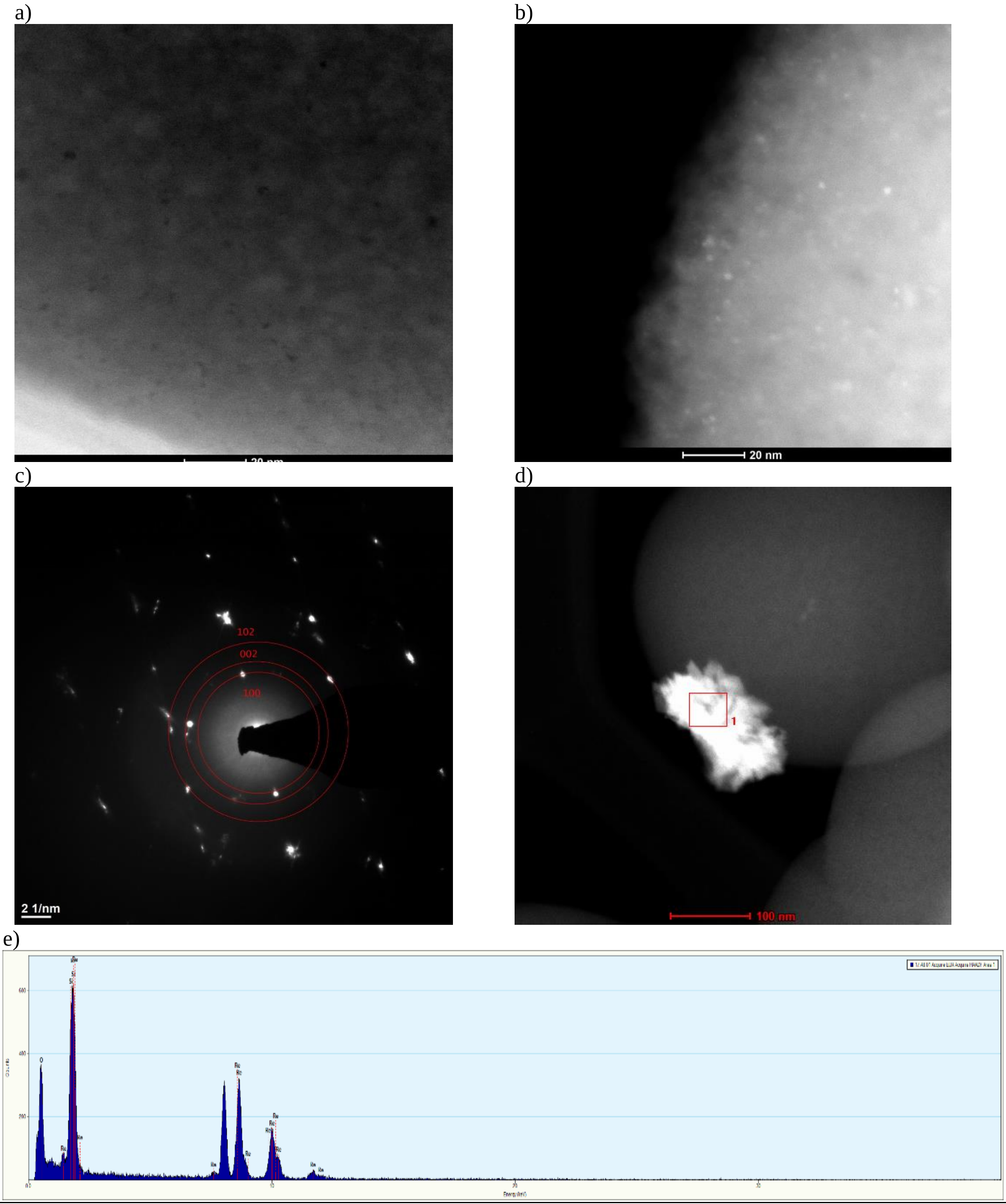

				Detector	
Element	Weight %	Atomic %	Uncert. %	Correction	k-Factor
-------	--------	--------	---------	----------	--------
O(K)	22.67	61.43	0.38	0.51	1.921
Si(K)	15.68	24.20	0.33	0.92	1.000
Re(L)	61.64	14.35	0.64	0.99	3.747

Fig. S6. Results of transmission electron microscopy analysis of Re nanoparticles: a) STEM-BF image, b) HAADF image, c) SAED diffraction images of selected Au particles, d,e) chemical composition analysis obtained using the characteristic EDS X-ray dispersion spectroscopy obtained in the areas indicated in the microscopic image (d)

a)

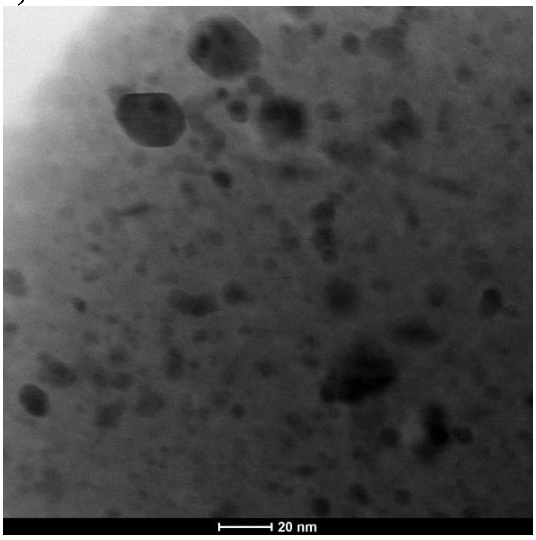

b)

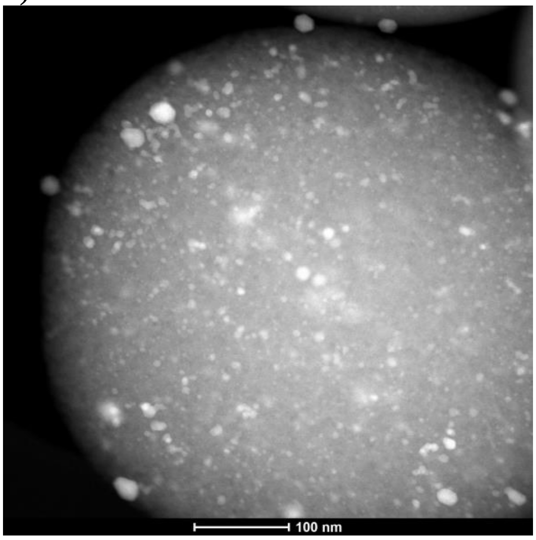

e)

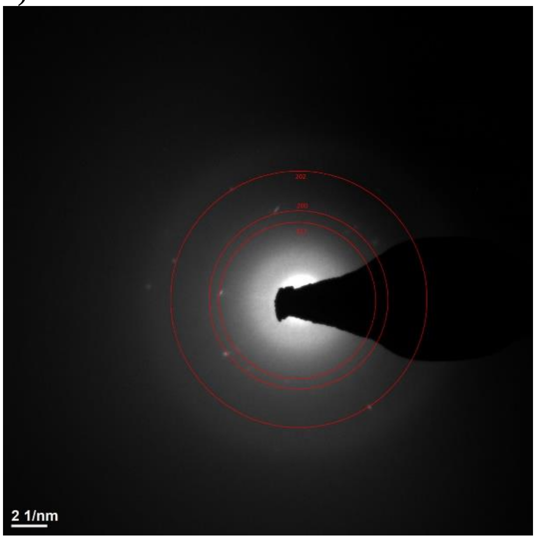

d)

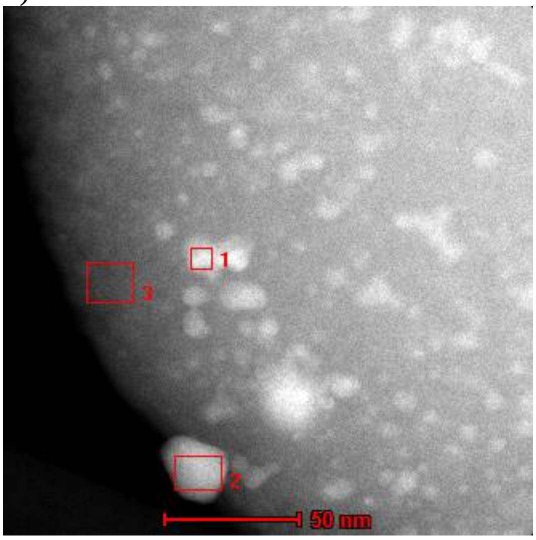

e)

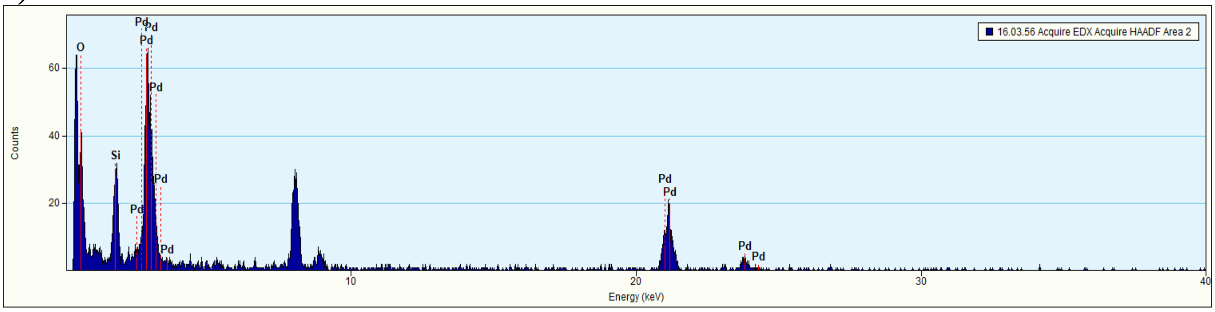

Detector					
Element	Weight %	Atomic %	Uncert. %	Correction	k-Factor
-------	--------	--------	---------	----------	--------
O(K)	24.24	61.29	1.13	0.51	1.921
Si(K)	9.33	13.44	0.52	0.92	1.000
Pd(K)	66.42	25.25	2.70	0.92	5.849

Fig. S7. Results of transmission electron microscopy analysis of Pd nanoparticles: a) STEM-BF image, b) HAADF image, c) SAED diffraction images of selected Au particles, d,e) chemical composition analysis obtained using the characteristic EDS X-ray dispersion spectroscopy obtained in the areas indicated in the microscopic image (d)

a)

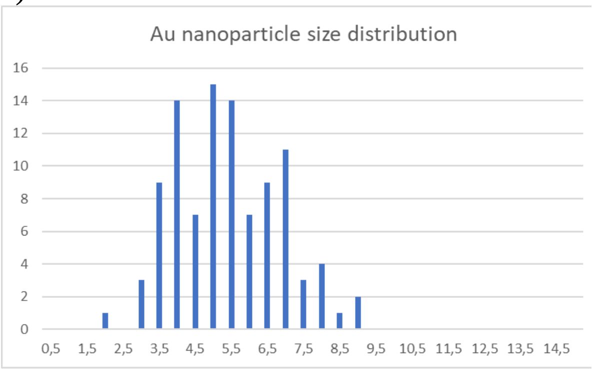

b)

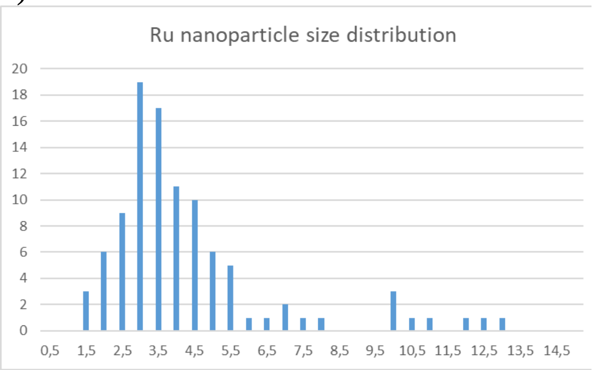

c)

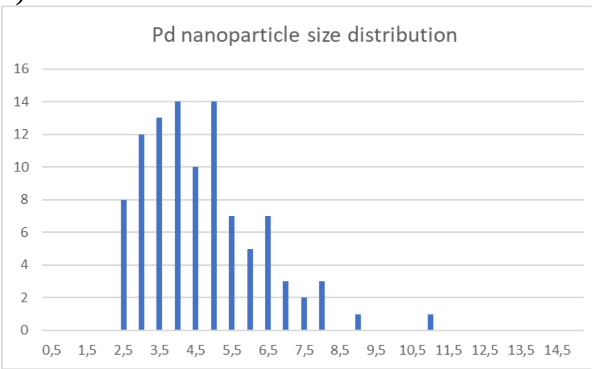

d)

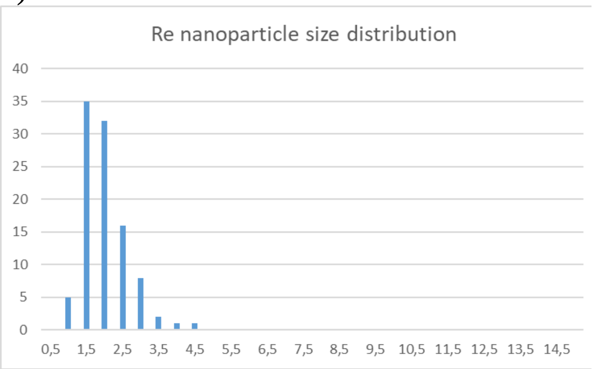

Fig. S8. Comparison of the particle size distribution obtained from TEM of analysed samples containing: a) Au, b) Ru, c) Pd and d) Re nanoparticles

Observations of the microstructures of both the ceramic preforms and composite materials were made using a Cs-corrected transmission electron microscope S/TEM Titan 80-300 from FEI company (Eindhoven, The Netherlands). Scanning transmission electron microscopy (STEM) and high-resolution transmission electron microscopy (HRTEM) imaging were also used as primary research techniques. The diffraction patterns were obtained with both selected area diffraction (SAED). STEM images using BF (bright field) confirms good dispersion of the synthesized nanoparticles was confirmed, which, with the exception of Re, rarely form agglomerates. Using SAED diffraction images and unit cell simulations, it was confirmed that the obtained nanoparticles are single-phase metals regarding the data collected in the table below. Comparison of the particle size distribution obtained from TEM of analysed samples containing different nanoparticels is shown on Fig. S8. The average particle size measured by transmission electron microscopy was 4.1nm for Ru, 4.4nm for Pd, 5.1nm for Au and 1.8nm for Re.

Au – Fig. S4c

Structural formula: AuChemical formula (sum): AuSystematic name: Gold - 3CCrystal structure data from: J. Spreadborough, J. W. Christian (1959) High-temperature X-ray diffractometer. Journal of Scientific Instruments 36:116-118

Structure Type: Crystal
Chemical Formula: Au
Z: 1
Space Group: F m $\bar{3}$ m
Crystal System: Cubic
a: 4.0700(100) Å
Cell Volume: 67.419 Å^3
Asymmetric Unit: 1 site
Unit Cell: 4 sites/unit cell
Density: 19.4087 g/cm^3
Visible Sites: 14

Ru – Fig. S5c

Crystal structure data from: Wyckoff, R. W. G. (1963) Second edition. Interscience Publishers, New York, New York Hexagonal closest packed, hcp, structure. Crystal Structures 1:7 - 83.

Structure Type: Crystal
Chemical Formula: Ru
Z: 1
Space Group: P 63/m m c
Crystal System: Hexagonal
a: 2.7039 Å
c: 4.2817 Å
Cell Volume: 27.110 Å^3
Asymmetric Unit: 1 site
Unit Cell: 2 sites/unit cell
Density: 12.3855 g/cm^3
Visible Sites: 2

Re – Fig. S6c

Crystal structure data from: Haglund, J., Fernandez Guillermet, F., Grimvall, G., Korling, M. (1993) Theory of bonding in transition-metal carbides and nitrides. Physical Review, Serie 3. B - Condensed Matter (18,1978-) 48:11685 - 11691.

Structure Type: Crystal
Chemical Formula: Pd
Z: 1
Space Group: F m $\bar{3}$ m
Crystal System: Cubic
a: 3.9000 Å
Cell Volume: 59.319 Å^3
Asymmetric Unit: 1 site
Unit Cell: 4 sites/unit cell
Density: 11.9141 g/cm^3
Visible Sites: 14

Pd – Fig. S7c

Crystal structure data from: Shiraki, Koichi, Tsuchiya, Taku, Ono, Shigeaki (2003) Structural refinements of high-pressure phases in germanium dioxide. Acta Crystallographica Section B 59:701 - 708.

Structure Type: Crystal
Chemical Formula: Re
Z: 1
Space Group: P 63/m m c
Crystal System: Hexagonal
a: 2.6029(16) Å
c: 4.1620(90) Å
Cell Volume: 24.420 Å^3
Asymmetric Unit: 1 site
Unit Cell: 2 sites/unit cell
Density: 25.3231 g/cm^3
Visible Sites: 2

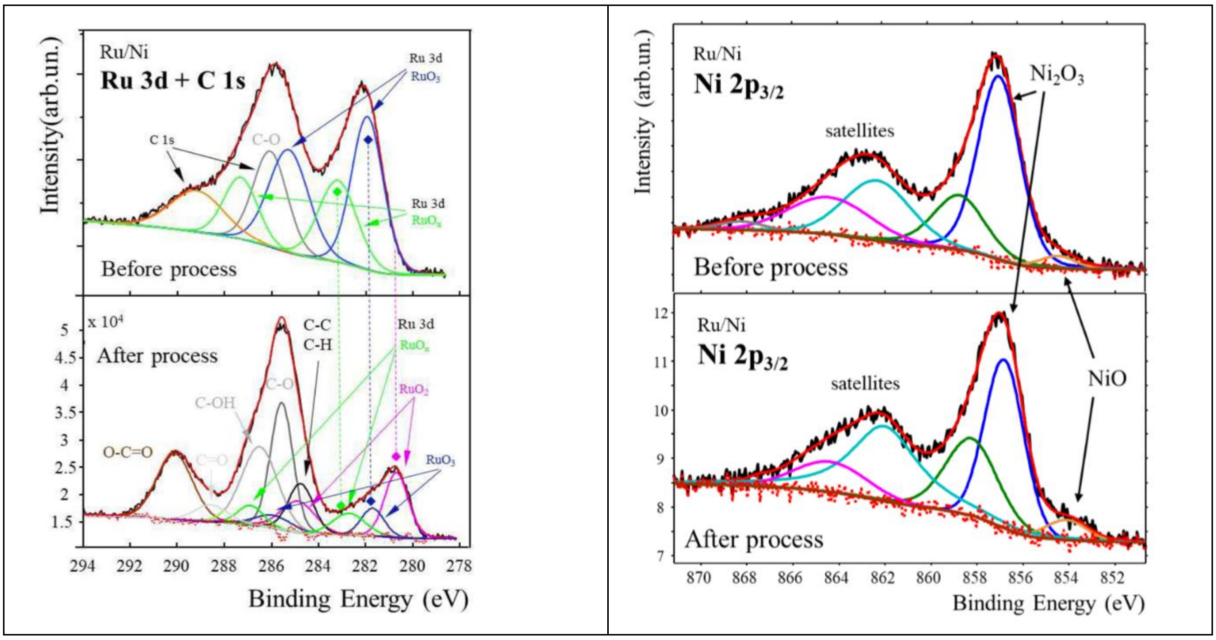

Fig. S9. XPS result for 1%Ru/Ni-wool before and after CO_2 methanation.

Physical Electronics' PHI 5700 spectrometer was used to perform X-ray photoelectron spectroscopy (XPS) on the Ni-based bimetallic composition. Monochromatic AlK_α x-ray radiation (hν = 1486.7 eV) was used to obtain the photoelectron spectra of core levels of particular elements. The structure of the obtained XPS multiplets was analyzed with the use of Multipak v. 9.0softwarefrom Physical Electronics.

For the best composition – 1%Ru/Ni-wool, the XPS spectrum before and after CO_2 methanation is shown. Analysis of the chemical states of Ni (see high resolution deconvolute XPS spectra of Ni $2p_{3/2}$) before and after CO_2 methanation indicates the presence of NiO and Ni_2O_3. A slight increase in NiO oxide was observed after the reaction, suggesting a partial valence transition of Ni^{2+} to Ni^{3+}. The presence of hydrogen (most likely in $Ni(OH)_2$, not found by XPS) is the most likely factor for such a transition. The Ru 3d and C 1s doublet photoemission lines, which have some energy range overlap, have been examined. The primary doublet at 281.8 (Ru $3d_{5/2}$) and 286.0 (Ru $3d_{3/2}$) eV reveals the highly oxidized RuO_3, which dominates the Ru component prior to the reaction. The origin of the second doublet of higher binding energy with the Ru $3d_{5/2}$ peak at 283.1 eV is unclear. It is likely that it comes from a complex oxide or hydroxide structure where Ru has a valence of +6 or +7. Several chemical states can then be attributed to the lines coming from carbon. Before the reaction, carbon species have been detected on the sample surface. C–H or C–C (C–C and C–H forms cannot be differentiated by XPS) is a common state for both series of samples. This event may indicate that the system is active enough to bind ambient CO_2, which is further reduced during the catalyst hydrogen treatment step. The relative ruthenium-to-carbon ratio, which may be determined from the fitted XPS spectra attributed to Ru and C, is altered by methanation. After the reaction, the Ru/C ratio dropped from 2.7 to 0.7. The XPS analysis of carbon bonds revealed that the reaction also altered their structure. After methanation, a considerable increase in the contribution from carbon bonding to oxygen was seen, and a reasonably high-intensity peak at 290.0 eV, corresponding to a C=O, was seen in the sample.
